# Microbial BMAA and the Pathway for Parkinson’s Disease Neurodegeneration

**DOI:** 10.3389/fnagi.2020.00026

**Published:** 2020-02-07

**Authors:** Daniela Nunes-Costa, João Duarte Magalhães, Maria G-Fernandes, Sandra Morais Cardoso, Nuno Empadinhas

**Affiliations:** ^1^CNC–Center for Neuroscience and Cell Biology, University of Coimbra, Coimbra, Portugal; ^2^Ph.D. Programme in Biomedicine and Experimental Biology (PDBEB), Institute for Interdisciplinary Research, University of Coimbra, Coimbra, Portugal; ^3^Institute of Cellular and Molecular Biology, Faculty of Medicine, University of Coimbra, Coimbra, Portugal; ^4^Institute for Interdisciplinary Research (IIIUC), University of Coimbra, Coimbra, Portugal

**Keywords:** Parkinson’s disease, mitochondrial dysfunction, innate immunity, neurodegeneration, microbial β-*N*-Methylamino-L-alanine (BMAA)

## Abstract

The neurotoxin β-*N*-methylamino-L-alanine (BMAA) is a natural non-proteinogenic diamino acid produced by several species of both prokaryotic (cyanobacteria) and eukaryotic (diatoms and dinoflagellates) microorganisms. BMAA has been shown to biomagnify through the food chain in some ecosystems, accumulating for example in seafood such as shellfish and fish, common dietary sources of BMAA whose ingestion may have possible neuronal consequences. In addition to its excitotoxic potential, BMAA has been implicated in protein misfolding and aggregation, inhibition of specific enzymes and neuroinflammation, all hallmark features of neurodegenerative diseases. However, the exact molecular mechanisms of neurotoxicity remain to be elucidated in detail. Although BMAA is commonly detected in its free form, complex BMAA-containing molecules have also been identified such as the paenilamicins, produced by an insect gut bacterial pathogen. On the other hand, production of BMAA or BMAA-containing molecules by members of the human gut microbiota, for example by non-photosynthetic cyanobacteria, the Melainabacteria, remains only hypothetical. In any case, should BMAA reach the gut it may interact with cells of the mucosal immune system and neurons of the enteric nervous system (ENS) and possibly target the mitochondria. Here, we review the available evidence and hint on possible mechanisms by which chronic exposure to dietary sources of this microbial neurotoxin may drive protein misfolding and mitochondrial dysfunction with concomitant activation of innate immune responses, chronic low-grade gut inflammation, and ultimately the neurodegenerative features observed across the gut-brain axis in Parkinson’s disease (PD).

## Chronic Exposure to BMAA

Microbial metabolism is an endless source of compounds with diverse biological activities ranging from vitamins to toxins, with a tremendous impact on human health and disease. The neurotoxin β-*N*-methylamino-L-alanine (BMAA) is an example of a microbial compound to which humans are differently exposed but whose health impacts are still not fully understood, even though current evidence points to an association between BMAA exposure and susceptibility to neurogenerative diseases.

Crucial to understanding how humans may be chronically exposed to BMAA and thus to evaluate how they may be more susceptible to neurodegenerative diseases is identifying its natural producers and how this neurotoxin moves through the food chain. On the island of Guam, symbiotic *Nostoc* cyanobacteria associated with the roots of cycad plants have historically been considered the main source of BMAA in the ecosystem. The hypothesis, in its most recent formulation, states that BMAA is produced by cycad-associated cyanobacteria, accumulates in cycad seeds consumed by fruit bats or flying foxes that were part of the local population’s diet, or directly used to make flour, thus biomagnifying in the food chain. According to this hypothesis, chronic exposure to BMAA through the ingestion of contaminated plants and animals would, therefore, be a contributing factor in the development of the ALS-PDC pathology in Guam residents (Bradley and Mash, 2009). High-incidence clusters of this condition have also been described in populations from the Kii peninsula of Japan and from western New Guinea, both genetically distinct from each other and from the Guam population but also with documented use of cycad-derived products, supporting an environmental etiology (Ince and Codd, 2005). Elsewhere, contaminated fish and shellfish have been proposed as a significant source of human exposure through biomagnification of BMAA produced not only by marine cyanobacteria but also by dinoflagellates and diatoms (Brand et al., 2010; Jonasson et al., 2010; Banack et al., 2014; Jiang et al., 2014; Lage et al., 2014). A few studies have investigated if crops irrigated with BMAA-contaminated water can bioaccumulate BMAA, and although this seems to be the case under laboratory-controlled conditions, the only study that conducted a field experiment with BMAA-containing water from a cyanobacterial bloom found no BMAA accumulation in the irrigated vegetables (Contardo-Jara et al., 2014, 2018; Esterhuizen-Londt and Pflugmacher, 2019). Thus, the BMAA exposure risk associated with the consumption of vegetables irrigated with cyanobacterial bloom-contaminated water remains to be demonstrated.

Cyanobacteria are widespread producers of BMAA, as shown by several studies employing highly selective analytical methods that confirmed the presence of BMAA in lab-maintained cyanobacterial cultures of different genera belonging to sections I (*Chroococcus*, *Merismopedia*, *Mycrocystis*, *Synechocystis*), II (*Myxosarcina*), III (*Leptolyngbya*, *Lyngbya*, *Oscillatoria*) and IV (*Anabaena*, *Nostoc*; Banack et al., 2007; Li et al., 2010; Spáčil et al., 2010; Downing et al., 2011; Berntzon et al., 2013; Combes et al., 2013; Jiang et al., 2013; Lage et al., 2016a; Monteiro et al., 2017; Metcalf et al., 2017; Violi et al., 2019). Although cyanobacteria are photoautotrophic bacteria, a nonphotosynthetic clade of divergent cyanobacteria named Melainabacteria was identified in groundwater, tap water, wastewater treatment plants and also as members of the human gut microbiota (Di Rienzi et al., 2013; Soo et al., 2014). The production of BMAA by members of the gut microbiota would be another possible route of chronic exposure but this hypothesis has not been investigated thus far (Brenner, 2013). However, the fact that the BMAA biosynthetic genes have not been identified in any of its natural producers prevents further speculation on whether Melainabacteria share the BMAA biosynthetic machinery with their photosynthetic counterparts. Indeed, and despite the ever-increasing interest in BMAA, the biosynthetic origins of this nonproteinogenic amino acid remain elusive and the proposed metabolic routes are still hypothetical (Brenner et al., 2003; Downing and Downing, 2016; Nunn and Codd, 2017). Recently, it was argued that cyanobacterial BMAA may originate from the hydrolysis of a methylated peptide (Nunn and Codd, 2017) and indeed, BMAA occurs in nature in a bound form in at least one family of secondary metabolites, the paenilamicins (Müller et al., 2014). These peptides are produced by the honey bee intestinal pathogen *Paenibacillus larvae* by a hybrid nonribosomal peptide/polyketide synthase (NRPS/PKS) and the genetic basis for the synthesis of the two BMAA moieties has been elucidated (Müller et al., 2014). Notably, homologs of the NRPS modules responsible for the BMAA moieties could be detected in some bacterial genera, none belonging to the phylum Cyanobacteria. Still, the possibility that free BMAA can originate in some organisms through the breakdown of more complex molecules such as the paenilamicins deserves further investigation.

## The Gut-Brain Axis

Humans are constantly exposed to a wide variety of microbial metabolites including BMAA and the gastrointestinal (GI) tract represents the main interface, not only because it is in direct contact with microbial metabolites present in food and drinking water, but also because it is the most heavily colonized surface housing metabolically-active microorganisms at least as numerous as human cells. This enormous amount and diversity of microbes contributes with a giant genomic blueprint far superior to that of the human genome, and its products, together with dietary metabolites, have a pronounced impact on human biochemistry and physiology (Ley et al., 2006; Sekirov et al., 2010; Shibata et al., 2017). Intestinal microbes are known to be essential for the development and functionality of the host’s immune responses, for the regulation of gut motility, for maintenance of the intestinal barrier integrity and nutrient absorption, for the metabolism of therapeutic drugs, and prevention over inadvertent environmental exposure to toxic compounds (Sekirov et al., 2010; Cryan and Dinan, 2012). An imbalance in the normal microbiota elicited by diet, GI infection, stress or antibiotics, can significantly alter microbiome homeostasis with negative impacts on health (Stecher and Hardt, 2008; Claesson et al., 2012; Collins et al., 2012; Cryan and Dinan, 2012; Lee and Hase, 2014). In this article, we focus on the microbial-produced neurotoxin BMAA discussing how chronic exposure can affect the GI and nervous systems and lead to disease.

The human gut has its own nervous system, the enteric nervous system (ENS), a vast and complex network of neurons and glial cells within the bowel, grouped into ganglia located in the myenteric and the submucosal plexuses (Gershon, 1999; Lebouvier et al., 2009). The ENS depends extensively on the microbiota to develop neuronal electrophysiological properties, such as membrane action potential and sensory neuron excitability (McVey Neufeld et al., 2013), and it normally communicates with the central nervous system (CNS) *via* a bi-directional homeostatic route designated the gut-brain axis, involving neural, metabolic, hormonal and immunological signaling (Mayer, 2011; Collins et al., 2012; Cryan and Dinan, 2012). The vagus nerve is the main bidirectional pathway connecting the viscera to the brain (Forsythe et al., 2014). Microbial metabolites, namely neurotransmitters, can reach and affect brain development and functions through the gut-brain axis (Sekirov et al., 2010; Collins et al., 2012; Cryan and Dinan, 2012). Gut microbiota composition and dysbiosis also play a role in conditions of the CNS, including Parkinson’s disease (PD; Finegold et al., 2002; Parracho et al., 2005; Berer et al., 2011; Sampson et al., 2016).

## Gastrointestinal Disorders and Neurodegeneration: The Case of PD

PD is usually defined as a movement disorder characterized by motor symptoms, such as tremor, postural imbalance, bradykinesia and rigidity (Obeso et al., 2010). The histopathological postmortem hallmark of PD is the presence of α-synuclein-containing (ASYN) insoluble fibrous aggregates, termed Lewy bodies (LBs), accompanied by the progressive loss of dopaminergic neurons in the substantia nigra pars compacta (SNpc) in the CNS. Major causative gene mutations in familial PD are linked to proteins involved in mitochondrial metabolism and dynamics (Cardoso et al., 2005), which highlights a key role of mitochondria in the PD neurodegenerative process.

Sporadic PD has a long prodromal period during which several other features develop, namely olfactory impairment, sleep disturbances and depression (Reichmann et al., 2009). Additionally, GI dysfunction that includes dysphagia, gastroparesis and severe constipation (Wingate, 1999; Pfeiffer, 2003; Rao and Gershon, 2016) may precede the onset of motor symptoms by many years (Savica et al., 2009; Cersosimo and Benarroch, 2012). LBs were found in both the myenteric plexus and submucosal plexus neurons of postmortem PD patients (Wakabayashi et al., 1988; Braak et al., 2006) and even in presymptomatic cases (Braak et al., 2006). Indeed, accumulating evidence obtained from biopsy studies confirmed the presence of LBs in organs innervated by the vagus nerve such as the salivary glands, stomach, duodenum, colon and in the rectum (Cersosimo, 2015), indicating that the vagus nerve is the obvious route through which the disease spreads between the gut and the brain, leading to the hypothesis that a toxin or pathogen can trigger the disease (Hawkes et al., 2007; Brenner, 2013; Alam et al., 2017). Braak et al. (2003) showed that the progression of LBs, the PD histopathological marks, is in agreement with the hypothesis that a pathogen in the GI tract may trigger abnormal ASYN processing and then spread *via* retrograde axonal vagus transport from the ENS to the CNS and that the GI symptoms observed in the vast majority of PD patients are pre-motor manifestations of PD. Notably, ASYN fibrils injected in mice duodenum were able to propagate to the brain months after injection (Van Den Berge et al., 2019) with propagation occurring through the vagus since it could be inhibited by vagotomy (Kim et al., 2019). Indeed, individuals that underwent full truncal vagotomy had a decreased risk for subsequent PD, strongly implicating the vagus nerve in PD pathogenesis and again corroborating the involvement of an enteric pathogen or toxin (Svensson et al., 2015).

## Amino Acid Misincorporation, Protein Folding and Neurodegeneration

Neurodegenerative disorders share similar histopathological hallmarks of protein misfolding and aggregation, even though the protein component and affected brain region may differ (Ross and Poirier, 2004). These neurodegenerative disorders are mostly sporadic without a known etiological factor, with aging being the most relevant risk factor. Although most proteins encoded in the human genome can be removed by cellular quality systems when damaged, some polypeptides are prone to misfold and aggregate into toxic oligomers. Interestingly, these protein aggregates can further downregulate the activities of proteolytic pathways since they are protease-resistant. Thus, accumulation of protein aggregates in different neurodegenerative diseases and disruption of translational fidelity in terminally differentiated post-mitotic neurons are potential pathways leading to cell death (Lee et al., 2006).

Hundreds of amino acids are known to occur in nature although only 22 are used in protein synthesis. Some of those non-proteinogenic amino acids can be mistakenly charged onto tRNA and misincorporated into proteins, potentially causing loss of function and/or misfolding (Bullwinkle et al., 2014). Primary metabolites such as ornithine, homoserine, and homocysteine are substrates for aminoacyl-tRNA synthetases, but cellular quality control mechanisms can detect their mischarging onto tRNA and prevent their inclusion into proteins. Other non-proteinogenic amino acids, though, are secondary metabolites produced by certain microbial species and can elude quality control pathways of nonproducing organisms, behaving as toxins (Bullwinkle et al., 2014). It has been speculated that these dietary or microbial-produced toxic amino acids could, in addition to host genetic factors, represent an environmental trigger for the onset of neurodegeneration (Rodgers, 2014).

Accumulating evidence suggests that this is the case for BMAA, which has been reported to be misincorporated into human proteins in place of serine, resulting in protein misfolding and aggregation, ER stress and apoptosis (Dunlop et al., 2013; Glover et al., 2014; Main et al., 2016). In light of these observations, BMAA misincorporation into ASYN could be a possible mechanism of BMAA-induced neurodegeneration, since serine phosphorylation is heavily correlated with ASYN aggregation. It is conceived that the vast majority of ASYN present in Lewy body aggregates is phosphorylated at serine 129 (S129; Oueslati, 2016), however, the modulation of ASYN aggregation by S129 phosphorylation is still a matter of debate, since *in vivo* studies demonstrate that this association is far more complex than what may appear (McFarland et al., 2009). Nonetheless, BMAA misincorporation in this position could provoke an imbalance in the extension of S129 phosphorylation, thereby promoting ASYN aggregation. Interestingly, S87 phosphorylation was found to inhibit ASYN aggregation and the substitution of serine by BMAA in this position could hypothetically reshape the sequence milieu and therefore reduce phosphorylated S87, increasing the propensity of ASYN to aggregate (Oueslati et al., 2012).

While the observation that BMAA interacts very strongly with proteins causing their misfolding has not been disputed, some groups have failed to detect incorporation of BMAA into the primary structure of proteins thus calling into question the role of the misincorporation hypothesis in explaining BMAA’s observed neurotoxic effects (Beri et al., 2017; Onselen et al., 2017). Subsequently, alternative mechanisms have been investigated and BMAA has been found to strongly associate with melanin, to selectively inhibit the activity of certain enzymes and to interfere with and disrupt protein refolding *in vitro* by associating with proteins through electrostatic interactions strong enough to resist TCA precipitation and subsequent washing with SDS or DTT (van Onselen and Downing, 2018). In particular, the interaction of BMAA with melanin and neuromelanin, which seem to have an important role in neurotoxin sequestration and neurodegeneration, as well as its ability to inhibit human catalase, an enzyme directly involved in the detoxification of β-amyloid-linked cellular toxicity, have recently been proposed to also contribute to BMAA’s neurotoxicity (Delcourt et al., 2018; van Onselen and Downing, 2019). Whether BMAA causes proteins to misfold due to eventual misincorporation into their primary structure or through strong electrostatic interactions with nascent chains remains to be confirmed (Dunlop and Guillemin, 2019).

As indicated above, supporting evidence for BMAA’s putative role in triggering neurodegenerative diseases comes from the island of Guam, where endemic ALS-PDC has been linked to chronic exposure to BMAA in the diet of Guam residents (Bradley and Mash, 2009). Other factors have been proposed to be involved in the etiology of Guam ALS-PDC, namely secondary hyperparathyroidism due to low ingestion of calcium and magnesium together with high aluminum consumption, since deposits of these substances were observed in the brains of affected Guam residents (Garruto et al., 1984). Although we cannot fully discard this as a potential contributing factor, a study failed to prove altered levels of calcium, magnesium and heavy metal levels in patient blood, urine and hair. Parathyroid hormone and serum phosphorus levels—indicators of secondary hyperparathyroidism—were also reported to be within the normal range in Guam affected people (Ahlskog et al., 1995), rendering the heavy metal hypothesis unlikely to be the principal cause of endemic ALS-PDC. In addition, there is no report of secondary hyperparathyroidism treatment being successful in ALS-PDC patients. Nonetheless, BMAA was detected in brain proteins from PD, AD, and ALS patients but not in controls (Dunlop et al., 2013), and was also recently detected in the CNS of *antemortem* human individuals, including in one ALS patient where it was suggested to act as a disease-potentiating agent together with other environmental factors (Berntzon et al., 2015). Moreover, rats treated with BMAA display ALS-like neurological impairment (de Munck et al., 2013). Other studies have, however, failed to confirm such results casting doubt on the selectivity and reliability of the analytical methods employed by other groups (Meneely et al., 2016). On the other hand, a study with vervet monkeys found that dietary BMAA triggers the formation of neurofibrillary tangles and Aβ plaques characteristic of ALS-PDC and Alzheimer’s disease, whereas the co-administration of L-serine was shown to have a neuroprotective effect (Cox et al., 2016). The latter finding is consistent with BMAA being a substrate for seryl-tRNA synthetase and suggests that inhibition of the proposed BMAA misincorporation into brain proteins by L-serine may be linked with prevention of BMAA’s neurodegenerative effects (Main et al., 2016). Intriguingly, conversion of L-BMAA into D-BMAA has been detected in the liver and in the brain of mice, bringing additional complexity into the already puzzling field of BMAA research (Metcalf et al., 2017). More recently, an *in vitro* study revealed that human liver hepatocyte and intestinal epithelial cell cultures are incapable of metabolizing BMAA, suggesting the absence of detoxification pathways that should protect against dietary exposure to this toxin (Downing et al., 2019).

## Mitochondrial-Driven Innate Immunity Activation: A Possible Link Between BMAA and Neurodegeneration

Neuronal loss is the ultimate consequence in the pathophysiology of neurodegenerative diseases, although the specificity of the neurons that degenerate is quite distinguishable. While in AD the entorhinal cortical and hippocampal neurons are the first to be affected, in PD and ALS the SNpc and the motor neurons are the first to perish, respectively (Martin, 1999). Despite these distinct features, neurodegenerative diseases share many common characteristics such as mitochondrial failure and marked proinflammatory profiles (Gan et al., 2018). It is also well established that in most neurodegenerative diseases, the activity of the electron transport chain (ETC) complexes are consistently impaired, along with aberrant mitochondrial dynamics, higher fragmentation, swelling with deformed *cristae* and increased reactive oxygen species (ROS) production (Johri and Beal, 2012; Hroudová et al., 2014; Muyderman and Chen, 2014). Furthermore, studies in post-mortem brain samples and analysis of cerebrospinal fluid (CSF) of AD, PD and ALS patients have demonstrated a sheer correlation between disease and up-regulation of several proinflammatory cytokines involved in the innate immune response (Stephenson et al., 2018). The cause of these striking neuroinflammatory events is very often intertwined with loss of neuronal mitochondria fitness. Upon a given damage, mitochondria expose danger-associated molecular patterns (DAMPs; Grazioli and Pugin, 2018), signals that are recognized by cellular mechanisms involving pattern recognition receptors (PRR) and which are divided into four families: toll-like receptors (TLR), Rig-1-like receptors (RLR), Nod-like receptors (NLR) and C-type lectin receptors (CLR). When activated by mitochondrial DAMPS, these PRR receptors can induce the upregulation of the NF-kB pathway, leading to the release of proinflammatory cytokines, such as TNFα and IL-1β, the latter through the activation of the NLRP3 inflammasome (Cardoso and Empadinhas, 2018). Moreover, mitochondrial ROS overproduction can also activate the inflammasome, thus amplifying the overall inflammatory response (Saint-Georges-Chaumet and Edeas, 2016).

Mitochondria are unique organelles with their origins tracing back to an ancient α-proteobacterial lineage (Hibbing et al., 2010) that grants them exclusive features such as their own DNA, an independent replication process, their own ribosomes, the mitoribosomes, and bacterial-type phospholipids such as cardiolipin (Cardoso and Empadinhas, 2018; Grazioli and Pugin, 2018). Bacterial populations compete for nutrients in many ways, one being the production of noxious substances that evolved to control or kill competitors. In fact, this is a common strategy used for the discovery of new potential antibiotics (Hibbing et al., 2010). Mitochondria’s resemblance with bacteria makes them natural targets of many microbial byproducts and, indeed, many examples of microbial substances targeting mitochondria have been identified. *Streptococcus pneumoniae*, for example, produces pneumolysin, a pore-forming substance that causes neuronal death by specifically targeting mitochondria (Braun et al., 2007). Some other toxins do not even require the actual presence of bacteria to promote mitochondrial failure. Although the molecular mechanisms are still to be explored, *Staphylococcus aureus* α-toxin and *Helicobacter pylori* vacuolating cytotoxin (VacA) can promote cytotoxicity through mitochondrial impairment (Arnoult et al., 2009). It should be noted here that BMAA may also have the ability to induce mitochondrial dysfunction (Beri et al., 2017). *In vitro* studies with NSC-34 cells, a cell line of motor neurons showed that BMAA elicited a pronounced decrease in oxidative phosphorylation, altered calcium homeostasis and exacerbated ROS production. In addition, treatment with BMAA dramatically decreased N2a neuronal cell line viability that was assessed by measuring the activity of the mitochondrial enzyme succinate dehydrogenase (SD; Takser et al., 2016). In brain samples from the population of the Kii peninsula of Japan, mitochondrial disruption has also been shown to occur leading to increased ROS production (Hata et al., 2018). Unfortunately, the mechanism of action remains elusive.

Recent findings have confirmed that, in addition to the deleterious effects on mitochondria, BMAA exposure was also associated with the proinflammatory profile observed in neurodegenerative diseases. BMAA administration to rats was able to reproduce the neuronal phenotype of ALS, concomitantly with a strong expression of proinflammatory cytokines (Michaelson et al., 2017). Remarkably, BMAA was able to promote the upregulation of Glial Fibrillary Acidic Protein (GFAP) and, consequently, an evident astrogliosis in a rat model of ALS-PDC (Cai et al., 2018). Also, BMAA triggered cytotoxic effects in a RAW246.7 cell line and in BV-2 microglial cells (Takser et al., 2016), which confers potential immunomodulatory capacity to this neurotoxin.

## Conclusion

Although considerable attention has been dedicated to BMAA research over the past decades, many aspects of this field remain uncertain. The fact that early analytical methods for BMAA detection were found to be unreliable hindered further progress (Faassen, 2014) and even with the implementation of more selective methods some conflicting results are still reported, particularly when it comes to assessing the BMAA biosynthetic potential of cyanobacterial strains. Such results increasingly suggest that BMAA production by cyanobacteria may be transient, inconsistent in laboratory cultures, and subject to fluctuations provoked by certain stimuli such as nitrogen availability (Downing et al., 2011; Monteiro et al., 2017). The fact is that the biological functions of this diamino acid in nature are still unknown. The few studies conducted on its natural producers have concluded that it interferes with nitrogen metabolism and have hypothesized an allelopathic role for BMAA, a common biological phenomenon by which metabolites produced by some organisms affect certain physiological traits of others (Lage et al., 2016b; Popova et al., 2018). In the case of the BMAA-containing paenilamicins, production of the compound was shown to impact competing bacterial populations in the larval gut thus confirming its allelopathic role (Müller et al., 2014). Although the ability of human gut microbiota to produce or be impacted by BMAA or BMAA-containing molecules is still unknown and warrants future investigation, there is little doubt that seafood and particularly marine bivalves can be a non-negligible source of dietary BMAA, which justifies further epidemiological studies to assess the real risk posed by their consumption (Lance et al., 2018).

Another topic warranting future investigation in this field is the identification of host genetic factors underlying individual predisposition to developing sporadic neurodegenerative diseases as a result of chronic BMAA exposure. Indeed, besides well-studied mutations that cause familial neurodegenerative diseases, some genetic polymorphisms have been associated with the susceptibility of their sporadic counterparts (Rocchi et al., 2003; Bras and Singleton, 2009). We propose that susceptible individuals with certain genetic polymorphisms, including in elements of the translational machinery (Bullwinkle et al., 2014), may more often misincorporate BMAA into aggregation-prone proteins whose accumulation in neurons may trigger the onset of neurodegeneration, initially in the ENS and ultimately in the CNS, owing to retrograde transport through the vagus nerve, or plasma, to the brain. Additionally, microbial metabolites can bypass the intestinal mucosa barrier due to increased permeabilization of the gut barrier, which is known to be increased in PD (Forsyth et al., 2011) and would facilitate BMAA toxicity. Aging accompanied with a decrease in the immune response (immunosenescence) often attributed to PD is greatly associated with a leaky gut (Nagpal et al., 2018). Also of note, a study performed by Dawson et al. (1998) demonstrated that male rats were more susceptible to BMAA-induced toxicity than females, a very interesting observation given that PD has a higher incidence in males than females.

**Figure 1:**
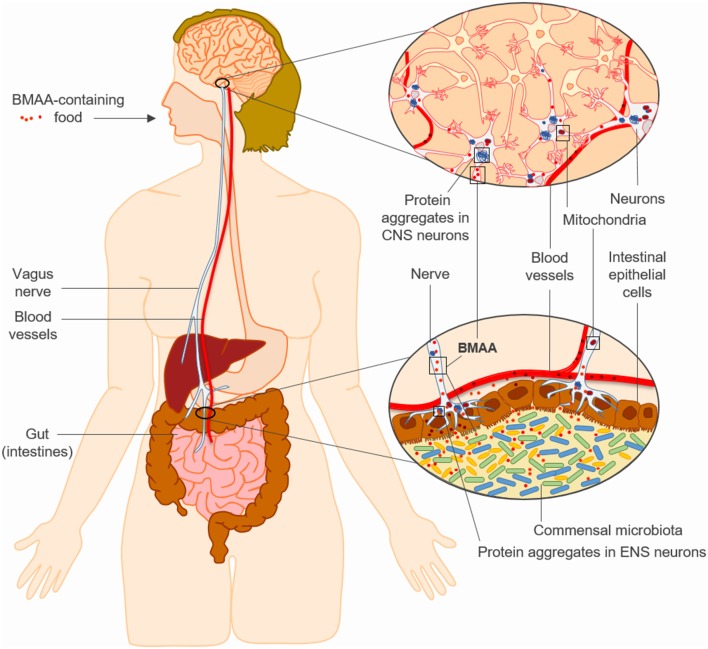
The “neurotoxin hypothesis” for sporadic neurodegenerative diseases such as Parkinson’s disease (PD). Chronic intestinal exposure to β-*N*-methylamino-L-alanine (BMAA) may trigger neurodegeneration by promoting protein misfolding, mitochondrial dysfunction and innate immune responses in genetically susceptible individuals, initially in the enteric nervous system (ENS) and later in the central nervous system (CNS) through retrograde transport *via* the vagus nerve.

In any case and as summarized in this review, BMAA effects in human cell lines and in animals are consistent with the hypothesis that chronic exposure to this neurotoxin can trigger features of sporadic neurodegenerative diseases such as PD (Figure 1). In this regard, it is reasonable to hypothesize that BMAA may promote protein misfolding, mitochondrial dysfunction and chronic innate immune activation in genetically susceptible individuals, initially in the ENS and later in the CNS by means of retrograde transport *via* the vagus nerve, and ultimately leading to brain neurodegeneration (Figure 1). The toxicological evidence from these studies is on its own sufficient to drive research towards the identification of the biosynthetic pathways and biological functions of this diamino acid in its natural producers, in-depth studies tracking BMAA through the food web in the various ecosystems, and epidemiological studies in populations exposed to BMAA-containing seafood. Only with this information, we will be able to accurately link the missing dots in the old but still controversial hypothesis of a BMAA-driven cause for sporadic neurodegenerative diseases in humans.

## Author Contributions

NE and SC: conceptualization. DN-C, JM, SC and NE: investigation. SC and NE: funding. DN-C, JM, MG-F, SC and NE: writing. 

## Conflict of Interest

The authors declare that the research was conducted in the absence of any commercial or financial relationships that could be construed as a potential conflict of interest. 
